# Supplementary material for: Influenza A Virus Infection Reactivates Human Endogenous Retroviruses Associated with Modulation of Antiviral Immunity
Source: Viruses. 2022 Jul 21;14(7):1591. doi: 10.3390/v14071591 (PMC9320126; doi:10.3390/v14071591)
Supplement: Supplementary file 1 [file viruses-14-01591-s001.zip › MotifAnalysis.zip!/MotifOutput_SC35MdNS1/homerResults/motif24.similar.html]

motif24

## Information for motif24

A
C
T
G
A
T
C
G
A
T
C
G
A
G
T
C
C
T
A
G
A
G
T
C
C
T
A
G
A
C
T
G
A
G
T
C
A
G
T
C
A
G
C
T
A
C
G
T
  
Reverse Opposite:  

C
G
T
A
C
T
G
A
A
C
T
G
A
C
T
G
A
G
T
C
G
A
T
C
C
T
A
G
A
G
T
C
T
C
A
G
A
T
G
C
A
T
G
C
T
G
A
C
  

|  |  |
| --- | --- |
| p-value: | 1e-10 |
| log p-value: | -2.436e+01 |
| Information Content per bp: | 1.758 |
| Number of Target Sequences with motif | 4.0 |
| Percentage of Target Sequences with motif | 5.19% |
| Number of Background Sequences with motif | 3.5 |
| Percentage of Background Sequences with motif | 0.01% |
| Average Position of motif in Targets | 117.2 +/- 39.1bp |
| Average Position of motif in Background | 99.1 +/- 25.3bp |
| Strand Bias (log2 ratio + to - strand density) | 2.0 |
| Multiplicity (# of sites on avg that occur together) | 1.00 |
| Motif File: | file (matrix) reverse opposite |

### Similar de novo motifs found

|  |  |  |  |  |  |  |  |
| --- | --- | --- | --- | --- | --- | --- | --- |
| Rank | Match Score | Redundant Motif | P-value | log P-value | % of Targets | % of Background | Motif file |
| 1 | 0.689 | G A C T A C T G A C T G A G T C A T G C A G C T G A C T G A C T | 1e-4 | -11.257133 | 15.58% | 3.45% | motif file (matrix) |
